# Investigating Peripheral *SIAH3* DNA Methylation in Adult Mental Disorders in Relation to Adverse Childhood Experiences

**DOI:** 10.3390/biom16070934

**Published:** 2026-06-23

**Authors:** Annika Bender, Laurine Schweizer, Mirac Nur Musaoglu, Sarah Pasche, Ariane Wiegand, Susanne Edelmann, Vanessa Nieratschker

**Affiliations:** 1 Department of Psychiatry and Psychotherapy, Eberhard Karls University of Tuebingen, 72076 Tuebingen, Germany; 2 German Center for Mental Health, Partner Site Tuebingen, 72076 Tuebingen, Germany; 3 Tuebingen Center for Mental Health (TüCMH), University Hospital Tuebingen, 72076 Tuebingen, Germany; 4 Max Planck Institute of Psychiatry, 80336 Munich, Germany; 5 German Center for Mental Health, Partner Site Munich-Augsburg, 80336 Munich, Germany

**Keywords:** DNA methylation, adult mental disorder, early adversity

## Abstract

Adult mental disorders (aMD), including borderline personality disorder (BPD), major depressive disorder (MDD), and social anxiety disorder (SAD), share adverse childhood experiences (ACEs) as an environmental risk factor. Epigenetic mechanisms, including DNA methylation (DNAm), may mediate the biological link between early adversity and psychiatric risk. *SIAH3*, implicated in stress-related and mitochondrial pathways, has been previously associated with both ACE and aMD. This study examined *SIAH3* DNAm in adults with BPD, MDD, or SAD, relative to healthy control participants (HC), testing effects of diagnosis, ACE exposure, and their interaction across the pooled sample and within each diagnostic group. Both aMD diagnosis and high ACE exposure showed trends toward *SIAH3* hypomethylation, and a significant diagnosis × ACE interaction emerged, with inconclusive post-hoc tests. Disorder-specific analyses revealed heterogeneous patterns: in BPD, high ACE showed a trend toward hypermethylation in unadjusted models; in MDD, interaction effects were marginal and not robust to covariate adjustment; in SAD, significant main effects and a diagnosis × ACE interaction were observed, with high ACE associated with lower DNAm exclusively in HC. These findings suggest disorder-specific epigenetic responses to ACE, positioning SIAH3 as a potential molecular link between early life stress, mitochondrial function, and aMD.

## 1. Introduction

Adverse childhood experiences (ACEs) are among the most potent environmental determinants of long-term mental health, with effects that extend to the epigenetic level [1,2,3]. Epigenetic modifications, including DNA methylation (DNAm), mediate long-term effects of experience on psychological and neurobiological development [4] and are increasingly studied in relation to psychiatric vulnerability [5,6,7]. Critically, DNAm may act as a biological mediator through which ACEs confer psychiatric risk, embedding environmental adversity into lasting molecular change. Mental disorders such as borderline personality disorder (BPD), major depressive disorder (MDD), and social anxiety disorder (SAD) strongly impair quality of life, with MDD and SAD being highly prevalent in adults (15.7%, 17%, respectively [8,9,10]). BPD involves instability in mood, self-image, behavior, and relationships [11]. MDD is characterized by persistent sadness, hopelessness, and reduced functioning [11]. SAD involves excessive fear of negative evaluation and social avoidance [11]. Although diagnostically distinct, these disorders often share similar environmental risk factors, notably ACE, which may contribute to overlapping deficits in emotion regulation, self-perception, and social functioning [12,13,14,15]. Despite their distinct clinical presentations, aMDs are increasingly understood within a transdiagnostic framework in which categorical diagnostic boundaries often reflect dimensional spectra of psychopathology rather than discrete biological entities [16,17]. Shared environmental risk factors, particularly ACE, alongside overlapping neurobiological and genetic vulnerabilities, support examining these disorders jointly to identify common epigenetic mechanisms of adversity, while within-disorder analyses preserve the ability to detect diagnostic moderation. Differential DNAm has been reported across BPD, MDD and SAD in genes linked to immune and inflammatory processes, cell cycling, signaling, and neuropsychiatric pathways [18,19,20,21,22], including genes encoding Brain Derived Neurotrophic Factor (*BDNF* [23,24,25]), Catechol-O-Methyltransferase (*COMT* [26,27,28]), and Monoamine Oxidase A (*MAOA* [29]).

Beyond these neurobiological targets, emerging evidence implicates mitochondrial dysfunction as a pathway through which ACE may confer psychiatric risk. Mitochondria regulate cellular energy metabolism, reactive oxygen species production, and stress-responsive signaling, i.e., processes central to neuronal integrity and allostatic regulation [30]. ACE has been associated with elevated mitochondrial copy number, a marker of mitochondrial stress [31,32], and mitochondrial dysfunction is increasingly recognized across multiple aMD [33], motivating investigation of genes at the intersection of epigenetic regulation and mitochondrial function. An emerging gene of interest is Seven in Absentia Homolog 3 (*SIAH3*), encoding an atypical E3 ubiquitin ligase that lacks a functional RING domain and differs from *SIAH1* and *SIAH2* in structure and function [34,35,36]. SIAH3 negatively regulates parkin RBR E3 ubiquitin protein ligase (PRKN) translocation to damaged mitochondria, possibly via PTEN induced kinase 1 (PINK1) inactivation [34,37], and modulates mitophagy and axonal degeneration [38,39]. It has also been implicated in neurodegenerative diseases [40] and cellular stress-related mechanisms [39,41]. A previous epigenome-wide association study (EWAS) reported *SIAH3* hypermethylation in individuals who later developed a mood disorder, although not surviving correction for multiple testing [42], and another identified *SIAH3* exon 2 hypomethylation associated with ACE, though not SAD diagnosis [43]. Furthermore, *SIAH3* expression was upregulated in mouse brains following maternal immune activation, a model of prenatal stress [44]. *SIAH3* was therefore selected as the candidate gene because it lies at the convergence of mitochondrial quality control, stress responsivity, and epigenetic regulation, and because prior research linked *SIAH3* DNAm variation to both ACE exposure and mood disorder onset [42,43,44].

While these previous findings point towards *SIAH3* as a biologically plausible locus through which ACEs may shape psychiatric risk via epigenetic mechanisms, it remains unknown whether ACE-associated differences in *SIAH3* DNAm are shared across BPD, MDD, and SAD, or whether they are disorder-specific and reflect divergent epigenetic responses to early adversity. The present study, therefore, examines ACEs as predictors of *SIAH3* DNAm across the combined sample and within each diagnostic group, testing whether the ACE-*SIAH3* DNAm relationship is transdiagnostic or moderated by diagnosis. Prior evidence on *SIAH3* DNAm directionality is limited and inconsistent: [42] reported prospective *SIAH3* hypermethylation as a risk marker for future mood disorder onset, a finding that did not survive correction for multiple testing, while [43] reported cross-sectional hypomethylation in association with ACE. Given this, directional predictions remain tentative. Based on the cross-sectional evidence most directly comparable to the present study [43], it was tentatively hypothesized that (i) aMD patients would show lower *SIAH3* DNAm compared to HC; (ii) high ACE exposure would be negatively associated with *SIAH3* DNAm; and (iii) the association between ACE and *SIAH3* DNAm would be moderated by diagnosis, potentially reflecting disorder-specific responses to early adversity.

## 2. Materials and Methods

### 2.1. Study Cohorts

All participants were European and aged 18–65 years. The total sample comprised 167 aMD patients and 187 healthy control participants (HC). The BPD cohort included 94 participants (*n* = 40 BPD patients, *n* = 53 HC) recruited as described in [45]. BPD diagnoses were based on the International Personality Disorder Examination (IPDE [46]) according to DSM-IV criteria [47]. The MDD cohort comprised 124 participants (*n* = 62 MDD patients, *n* = 62 HC) [48], with diagnoses established per DSM-IV by clinicians at the Department of Psychiatry and Psychotherapy, University Hospital Tuebingen (Germany). The SAD cohort included 137 participants (*n* = 65 SAD patients, *n* = 72 HC) [43]. Diagnoses were based on the Structured Clinical Interview for DSM-IV (SCID [49]). In all cohorts, HC had no history of psychiatric disorders. All participants provided written informed consent. The study was approved by the local ethics committee of the University of Tuebingen and conducted in accordance with the Declaration of Helsinki.

### 2.2. Measures

#### 2.2.1. Questionnaires

ACEs were assessed with the Childhood Trauma Questionnaire (CTQ), comprising five subscales (emotional and physical neglect, and emotional, physical and sexual abuse [50,51]). High ACE exposure was defined as a moderate or higher score on ≥1 subscale (sexual abuse: >8; physical abuse: >10; physical neglect: >10; emotional abuse: >3; emotional neglect: >15 [50,51]). Symptom severity was measured using disorder-specific instruments. For BPD, the Borderline Symptom List (BSL-23 [52]) was used, for MDD, the Beck Depression Inventory (BDI [53]) was employed, and for SAD, the Liebowitz Social Anxiety Scale (LSAS [54]) was utilized. For the BPD and MDD cohorts, additional symptom burden was captured by the Symptom Checklist-90-R (SCL-90-R [55]), including its three global scales: Global Severity Index (GSI), Positive Symptom Total (PST) and Positive Symptom Distress (PSDI).

#### 2.2.2. *SIAH3* DNAm Analysis in Saliva and Whole Blood

For the BPD cohort, whole blood and saliva samples were analyzed, while only whole blood samples were available for the MDD and SAD cohorts. Peripheral venous blood was collected in ethylenediaminetetraacetic acid (EDTA) tubes (SARSTEDT AG & Co. KG, Nümbrecht, Germany) and stored at −80 °C. Genomic DNA was isolated using the QIAmp DNA Blood maxi Kit (Qiagen, Hilden, Germany) following the manufacturer’s instructions. Saliva was collected with the OraGene^®^ DNA saliva kit (DNA Genotek, Ottawa, ON, Canada), stored at −80 °C, and processed with the prepIT L2P solution (DNA Genotek) per the manufacturer’s instructions. 500 ng of genomic DNA were bisulfite-converted using the EpiTect Fast Bisulfite Kit (Qiagen) and stored at −20 °C. For the SAD cohort, DNAm data for cg04924408 and cg03997626, adjusted for cell-type ratios, were obtained from [43]. A region on chromosome 13q14.13 (GRCh37/hg19: chr13: 46,356,348–46,356,489), located in exon 2 of *SIAH3*, was amplified (forward primer: 5′-GTTGGTTATTATTTATAAATTTGGGAGG-3′, reverse primer: 5′-Biotin-TCACTATATCATTCATACTACAACAAATACT-3′ (Metabion, Planegg/Steinkirchen, Germany)), spanning CpG1 (cg04924408; chr13: 46,356,395), CpG2 (unannotated; chr13: 46,356,410), and CpG3 (cg03997626; chr13: 46,356,409). DNAm levels were quantified in technical duplicates with ≤3% absolute difference using the PyroMark Q24 system (sequencing primer: 5′-TTAAAATGTTTTTATYGTTTTA-3′ (Metabion)) and the PyroMark Q24 software (version 2.0.7, Qiagen). Assay performance was validated by titration with standardized bisulfite-converted control DNA (EpiTect Control DNA, Qiagen).

### 2.3. Data Analyses

Differences in the sociodemographic and clinical data of patients and HC were assessed using the Wilcoxon rank-sum test or, for factorial variables, the Fisher’s exact test after non-normality was confirmed using the Shapiro-Wilk test. Statistical data analyses were performed using the software environment R (version 4.5.1). More detailed information on analyses is reported in the supplement. Briefly, for each participant, an *SIAH3* DNAm value was calculated by averaging across the technical replications and CpG sites assessed. Group differences in *SIAH3* DNAm were evaluated across the pooled aMD sample and within each cohort (BPD, MDD, SAD) to examine disorder-specific effects. *SIAH3* DNAm levels, expressed as positive continuous percentage values, were modeled by a generalized linear mixed-effects model (GLMM; for aMD) or generalized linear models (GLM; for separate cohort analyses) with a Gamma distribution and log link to account for skewed distributions, using the glmmTMB package [56]. Primary analyses adjusted for age, sex, and smoking status. Unadjusted models were included in the supplement as sensitivity analyses. Model fits were evaluated through visual inspection of residual and Q-Q plots. Where applicable, estimated marginal means (EMMs) were computed on the log scale following GLMM or GLM computation using the emmeans package [57]. The significance of fixed effects and, where applicable, their interaction was tested using a Type III Wald χ^2^ test of deviance, which was performed using the Anova() function of the car package [58]. Further, the proportion of variance explained by the fixed effects and marginal R^2^ values was computed using the MuMIn package [59]. Given the lognormal GLM structure, the marginal R^2^ for the lognormal model was reported, complemented by marginal means on the original scale with 95% confidence intervals (CIs). To verify transdiagnostic consistency, a cohort-stratified sensitivity analysis was conducted. Estimates of the GLMs of the individual cohort-level with covariate adjustment were pooled using fixed-effects inverse-variance weighting (IVW) on the log scale and back-transformed to effect ratios (*e*^β^) for comparison with the primary random intercept GLMM. Preliminary data inspection indicated grouping tendencies of *SIAH3* DNAm across cohorts and diagnoses, prompting exploratory kernel density estimation (KDE) analyses. First, Hartigan’s dip test was applied to the pooled aMD sample, BPD (blood and saliva), MDD and SAD cohorts using the diptest package [60]. For cohorts showing significant deviation from unimodality, density-based modes of mean DNAm were characterized. Kernel density estimates were computed for each cohort, and local maxima of the density functions were identified as candidate DNAm modes using the second derivative criterion. Each sample was assigned to its nearest mode based on minimal absolute distance, generating discrete DNAm modes for downstream analyses. Subsequently, Kruskal–Wallis tests followed by Dunn’s post hoc tests with Benjamini-Hochberg correction were used to identify variables associated with DNAm modes. Technical variables were included in this analysis, with “blood storage duration” indicating the storage time of blood samples until DNA extraction, and “DNA storage duration” referring to the duration from DNA extraction until analysis. To formally test whether pre-analytical variation accounted for observed multimodal distributions, KDE mode membership was regressed on blood storage duration and DNA storage duration as the only predictors using multinomial regression (Type II likelihood ratio tests). Further, cell-type adjusted DNAm values were computed [61] for a subset of the MDD cohort for which the data were available to additionally examine cell counts as potential drivers of the groupings. To quantify *SIAH3* transcript levels, transcriptomic data were derived from whole-blood RNA-sequencing protocols as previously reported [62] and specified in more detail in the supplement. To assess a potential association of *SIAH3* blood DNAm levels with cell-type-adjusted, DESeq2-normalized gene expression values, Spearman’s rank correlation was performed. Gene expression data were available for the SAD cohort only.

## 3. Results

The demographic and clinical, and all statistical test values of the pooled aMD sample, BPD, MDD and SAD cohorts, respectively, are displayed in Table 1.

### 3.1. SIAH3 DNAm Levels in aMD Patients and HC with Regard to ACE

**Figure 1:**
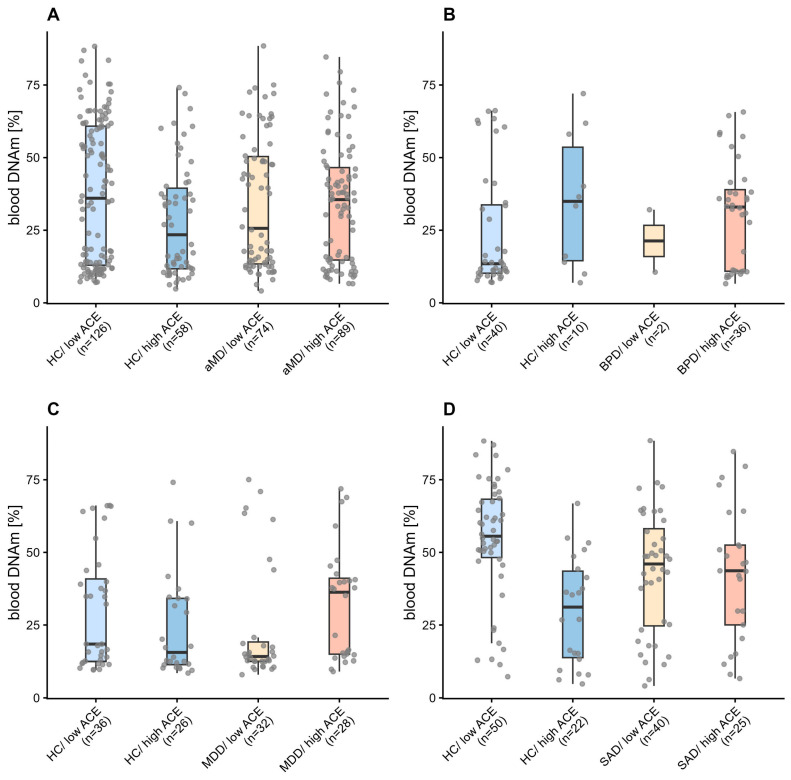
*SIAH3* blood DNAm across cohorts. *SIAH3* blood DNAm levels of all study participants are displayed with respect to diagnosis and ACE status ((**A**) pooled sample, patients indicated by aMD; (**B**) BPD cohort; (**C**) MDD cohort; (**D**) SAD cohort).

The epigenetic data of the pooled aMD sample, BPD, MDD and SAD cohorts are listed in Table 2. Figure 1 shows the *SIAH3* blood DNAm levels of all cohorts by diagnosis and ACE status (*SIAH3* saliva DNAm of the BPD cohort is displayed in Figure S1).

The GLMM fitted for the pooled aMD sample (*SIAH3* DNAm level ~ diagnosis × ACE + age + sex + smoking status + (1|Cohort), AIC = 2736.5, dispersion estimate = 0.423; R^2^ = 0.0307; predicted mean DNAm [%] [lower CI, upper CI]: HC low ACE: 34.0 [25.2, 45.9], HC high ACE: 27.5 [19.9, 38.0], aMD low ACE: 27.9 [20.3, 38.4], aMD high ACE: 35.1 [26.0, 47.4]) revealed a trend toward lower DNAm levels in patients compared with HC (β = −0.1967, SE = 0.1078, z = −1.83; χ^2^(1) = 3.3321, pANOVA = 0.0679) and a trend-level negative association of high ACE exposure with DNAm levels (β = −0.2114, SE = 0.1086, z = −1.95; χ^2^(1) = 3.7891, pANOVA = 0.0516). None of the covariates predicted *SIAH3* DNAm levels (Table S4). A significant diagnosis × ACE interaction was detected (β = 0.4403, SE = 0.1583, z = 2.78; χ^2^(1) = 7.7421, pANOVA = 0.0054), indicating differential associations of ACE with DNAm by diagnostic status; however, post hoc tests were non-significant (Table S5). The interaction was also observed in the unadjusted sensitivity analysis, where a further significant main effect of ACE was observed (Tables S6 and S7). Cohort-stratified sensitivity analysis demonstrated a directionally consistent negative main effect of diagnosis across all cohorts (Figure S2). Fixed-effects IVW meta-analytic pooling yielded a log-scale estimate of −0.26 (SE = 0.11); effect ratio 0.77 (95% CI: [0.62, 0.96]). This was directionally aligned with, although somewhat stronger than, the primary transdiagnostic random-intercept GLMM log-scale estimate of −0.20 (SE = 0.11); effect ratio 0.82 (95% CI: [0.67, 1.02]).

### 3.2. SIAH3 DNAm Levels in Patients and HC with Regard to ACE per Clinical Subgroup

In the BPD cohort, *SIAH3* blood DNAm did not differ by diagnosis (GLM: *SIAH3* blood DNAm level ~ diagnosis + age + sex+ smoking status, AIC = 742.7, dispersion estimate = 0.478, R^2^ = 0.0443, β = 0.0389, SE = 0.1806, z = 0.22; χ^2^(1) = 0.0463, pANOVA = 0.8296; predicted mean DNAm [%] [lower CI, upper CI]: HC: 29.0 [21.7, 38.6], BPD: 30.1 [23.1, 39.2]), and no covariates were associated with DNAm, with the sensitivity analysis reflecting these results (Tables S8 and S9). Neither ACE status nor any covariates were associated with blood DNAm in the adjusted model (GLM: *SIAH3* blood DNAm level ~ ACE + age + sex + smoking status, AIC = 741.6, dispersion estimate = 0.472, R^2^ = 0.0585, β = 0.2008, SE = 0.1816, z = 1.11; χ^2^(1) = 1.2228, pANOVA = 0.2688; predicted mean DNAm [%] [lower CI, upper CI]: low ACE: 25.6 [18.5, 35.5], high ACE: 31.3 [24.8, 39.6]), although unadjusted analysis suggested a trend-level positive association of ACE with DNAm (GLM: *SIAH3* blood DNAm level ~ ACE, AIC = 743.5, dispersion estimate = 0.476, R^2^ = 0.0480, β = 0.279, SE = 0.1473, z = 1.89; χ^2^(1) = 3.5884, pANOVA = 0.0582; predicted mean DNAm [%] [lower CI, upper CI]: low ACE: 23.9 [19.4, 29.5], high ACE: 31.6 [25.9, 38.6]; Tables S10 and S11). For *SIAH3* saliva DNAm, no effects of diagnosis (GLM: *SIAH3* saliva DNAm level ~ diagnosis + age + sex + smoking status, AIC = 769.0, dispersion estimate = 0.226, R^2^ = 0.0167, β = −0.0288, SE = 0.1186, z = −0.24; χ^2^(1) = 0.0590, pANOVA = 0.8081; predicted mean DNAm [%] [lower CI, upper CI]: HC: 39.9 [32.9, 48.3], BPD: 38.7 [32.4, 46.3]) or ACE status (GLM: *SIAH3* saliva DNAm level ~ ACE + age + sex + smoking status, AIC = 768.8, dispersion estimate = 0.226, R^2^ = 0.0190, β = 0.0546, SE = 0.1186, z = 0.46; χ^2^(1) = 0.2123, pANOVA = 0.6449; predicted mean DNAm [%] [lower CI, upper CI]: low ACE: 37.8 [30.5, 46.8], high ACE: 39.9 [33.9, 47.0]) were observed, as reflected in the respective unadjusted analyses (Tables S12–S15).

In the GLM of the MDD cohort (*SIAH3* blood DNAm level ~ diagnosis × ACE + age + sex + smoking, AIC = 1023.4, dispersion estimate = 0.412, R^2^ = 0.0849; predicted mean DNAm [%] [lower CI, upper CI]: HC low ACE: 26.6 [20.9, 34.0], HC high ACE: 23.1 [17.4, 30.5], MDD low ACE: 21.5 [17.0, 27.4], MDD high ACE: 29.4 [22.7, 38.0]), there was a significant positive association of smoking with *SIAH3* DNAm (β = 0.3279, SE = 0.1468, z = 2.23; χ^2^(1) = 4.9914, pANOVA = 0.0255), while all other covariates showed no effects (Table S16). Further, a trend-level interaction of diagnosis × ACE (β = 0.4549, SE = 0.2362, z = 1.93; χ^2^(1) = 3.7100, pANOVA = 0.0541) emerged, which reached significance only in the unadjusted sensitivity analysis (Table S17). Given the lack of robustness to covariate adjustment, post-hoc tests were not performed.

The GLM for the SAD cohort (*SIAH3* DNAm level ~ diagnosis × ACE + age + sex + smoking status, AIC = 962.6, dispersion estimate = 0.317, R^2^ = 0.1893; predicted mean DNAm [%] [lower CI, upper CI]: HC low ACE: 56.8 [46.5, 69.3], HC high ACE: 27.9 [21.2, 36.8], SAD low ACE: 41.5 [32.9, 52.4], SAD high ACE: 43.5 [34.1, 55.5]) revealed significant negative main effects of diagnosis (β = −0.3129, SE = 0.1432, z = −2.19; χ^2^(1) = 4.7764, pANOVA = 0.0289) and high ACE exposure (β = −0.7095, SE = 0.1654, z = −4.29; χ^2^(1) = 18.3975, pANOVA < 0.0001), as well as a significant diagnosis × ACE interaction (β = 0.7558, SE = 0.2306, z = 3.28; χ^2^(1) = 10.7439, pANOVA = 0.0010). Covariates did not influence *SIAH3* DNAm (Table S18). Post hoc tests (Table S19) indicated significantly lower DNAm levels among high ACE HC (*p* = 0.0001), whereas no such effect was observed in SAD patients. Among high ACE individuals, DNAm levels showed a trend-level positive association with SAD diagnosis (*p* = 0.0640). Sensitivity analyses yielded a comparable pattern (Tables S20 and S21), although the main effect of diagnosis was marginal (β = −0.2333, SE = 0.1215, z = −1.92; χ^2^(1) = 3.6867, pANOVA = 0.0548). Notably, a trend-level negative correlation between *SIAH3* blood DNAm and cell-type-adjusted gene expression values was observed across the SAD cohort (Spearman’s ρ = −0.1614, S = 497,712, *p* = 0.0595; Figure S3). Within-group sensitivity analyses showed a consistent negative direction in both HC and SAD patients (see Figure S3; median adjusted expression (IQR): HC low ACE: 2.16 (4.18); HC high ACE: 2.39 (2.63); SAD low ACE: 1.88 (2.79); SAD high ACE: 3.60 (3.07)).

### 3.3. Exploratory Analysis and Characterization of SIAH3 DNAm Modes

**Figure 2:**
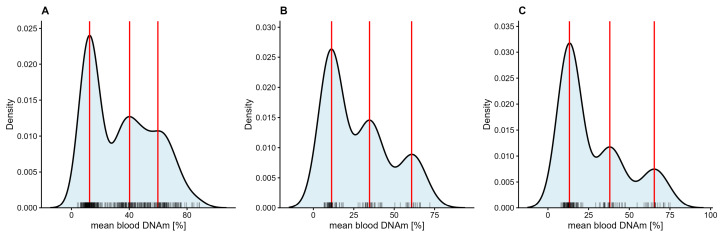
Exploratory DNAm mode density plots. *SIAH3* DNAm density plots with corresponding modes representing local density maxima (red) of the pooled aMD sample (**A**), BPD (**B**), and MDD (**C**) cohorts, including respective rug plots.

Hartigan’s dip test indicated significant deviation from unimodality in the pooled aMD sample (D = 0.0396, *p* = 0.0005), BPD blood (D = 0.0615, *p* = 0.0129), and MDD (D = 0.0468, *p* = 0.0455) cohorts, but not in the BPD saliva (D = 0.0451, *p* = 0.1895) or the SAD cohorts (D = 0.0357, *p* = 0.2444). Accordingly, exploratory KDE was performed for the pooled aMD sample, BPD blood and MDD cohorts to further characterize potential multimodality. In each cohort, three DNAm modes (modes 1–3, from lower to higher DNAm levels) were identified (Figure 2).

Full mode characterizations are provided in Table S22 (aMD), Table S23 (BPD blood), and Table S24 (MDD). In the pooled aMD sample, modes 2 and 3 differed significantly in DNAm mean and CpG1 and CpG2 DNAm (all adjusted *p* < 0.0001). Mode 2 contained the highest proportion of individuals with high ACE exposure (Mode1–Mode2 *p* = 0.0090; Mode2–Mode3 *p* = 0.0003). Further, modes 2 and 3 differed significantly in CTQ total score (*p* = 0.0012), and the subscales emotional (*p* = 0.0020) and sexual abuse (*p* = 0.0031), with the highest scores in mode 2, the lowest in mode 3, and intermediate values in mode 1. Modes 1 and 3 additionally differed in DNA storage duration (*p* = 0.0003), with mode 3 samples having the shortest duration. No mode-specific differences were observed for diagnosis, sex, age, smoking status, the CTQ subscales of emotional and physical neglect, and physical abuse, as well as for blood storage duration. Although only DNA storage duration showed significant univariate differences between modes, formal multinomial mode assignment models (with blood storage duration and DNA storage duration as only predictors) indicated that both pre-analytical variables significantly predicted mode membership (blood storage duration: χ^2^(2) = 9.0849, *p* = 0.0107; DNA storage duration: χ^2^(2) = 30.8774, *p* < 0.0001), necessitating restriction of exploratory KDE mode interpretation to within-cohort analyses.

In the BPD blood cohort, DNAm mean and CpG 1–3 DNAm differed significantly across all modes (all adjusted *p* of Mode1–Mode2 and Mode1–Mode3 < 0.0001; all adjusted *p* of Mode2–Mode3 comparisons 0.007). Modes 1 and 2 differed in the proportion of individuals with high ACE exposure (*p* = 0.0192) and BPD diagnosis (*p* = 0.0200), with mode 1 showing the lowest and mode 2 the highest proportions. Consistently, BSL-23 scores were highest in mode 2 and lowest in mode 1 (*p* = 0.0072), while sexual abuse scores were also highest in mode 2 (Mode1–Mode2 *p* = 0.0035; Mode2–Mode3 *p* = 0.0036). Mode 1 showed the shortest blood storage duration (Mode1–Mode2 *p* < 0.0001; Mode1–Mode3 *p* = 0.0114). No significant mode differences were detected for sex, smoking status, age, CTQ total score or remaining subscales, GSI, PST, or PSDI, or DNA storage duration. Since multinomial regression indicated that blood storage duration significantly predicted mode membership (χ^2^(2) = 7.9796, *p* = 0.0185), whereas DNA storage duration did not (χ^2^(2) = 0.0795, *p* = 0.9610), the observed BPD mode structure may at least partially reflect pre-analytical variation, and therefore, cautious interpretation of BPD multimodality is warranted.

In the MDD cohort, three distinct DNAm modes were likewise identified. All DNAm measures (DNAm mean and DNAm of CpG1–3) differed significantly between the modes (all adjusted *p* of Mode1–Mode2 and Mode1–Mode3 < 0.0001; Mode2–Mode3: DNAm mean *p* = 0.0160, DNAm CpG1 *p* = 0.0221, DNAm CpG2 *p* = 0.0086, DNAm CpG3 *p* = 0.0207), whereas none of the remaining assessed variables showed significant mode-specific differences. Multinomial regression showed that neither technical variable predicted mode membership (blood storage duration: χ^2^(2) = 3.0442, *p* = 0.2183; DNA storage duration: χ^2^(2) = 0.3813, *p* = 0.8262), indicating that the multimodal distribution in this cohort was not attributable to the pre-analytical variation assessed herein. In a subset of 94 participants of the MDD cohort (57 HC, 37 MDD patients), unadjusted and cell-type-adjusted DNAm values were strongly correlated (Spearman’s ρ = 0.8563, *p* < 0.0001), indicating high concordance between measures. Repetition of Hartigan’s dip test for the cell-type-adjusted subset, however, revealed assumed unimodality (D = 0.0294, *p* = 0.8522). Hartigan’s dip test applied to unadjusted DNAm values within this same *n* = 94 subset also did not reach significance (D = 0.0497, *p* = 0.0856), indicating that the multimodality signal is not detectable in this subsample regardless of cell-type adjustment. Notably, the blood cell counts (neutrophils, lymphocytes, and monocytes in percentages) did not differ across DNAm modes in the cell-type-unadjusted KDE analysis.

## 4. Discussion

This study investigated *SIAH3* DNAm patterns in aMD patients generally, as well as in BPD, MDD, and SAD patients separately, compared to HC, accounting for ACE exposure. Our main analysis revealed significant diagnosis × ACE interaction effects in the pooled aMD sample and SAD cohort, with a trend-wise effect in the MDD cohort. While the interaction was characterized by differential effects of ACE in HC but not in SAD patients in the SAD cohort, the interaction was inconclusive in the pooled aMD sample. The SAD cohort further revealed significant main effects of diagnosis and ACE, both negatively associated with *SIAH3* DNAm. Further analyses suggest non-uniform relationships across aMD. Overall, the tentative directional hypotheses were only partially supported. Diagnostic hypomethylation (hypothesis i) and a negative ACE effect (hypothesis ii) emerged at trend level in the pooled sample and more robustly in the SAD cohort, while the moderation of ACE effects by diagnosis (hypothesis iii) was confirmed in the pooled and SAD analyses, though the post-hoc structure of the interaction diverged from a straightforward ACE effect, with the effect concentrated in HC rather than patients.

In the pooled aMD sample, high ACE exposure was trend-wise associated with lower *SIAH3* DNAm. The significant diagnosis × ACE interaction resulted in inconclusive post hoc tests, while neither diagnostic status nor covariates predicted DNAm. To evaluate disorder-specific effects, each cohort was further analyzed separately. In the BPD cohort, characterized by high trauma prevalence among patients, a trend-level positive association of ACE with blood DNAm was observed only in unadjusted analyses, with no diagnostic effect detected. Neither were any saliva-based associations observed. Due to sample constraints, diagnosis × ACE interactions could not be reliably tested in this cohort. These potentially underpowered preliminary findings do not rule out potential ACE-related influences that warrant investigation in larger, balanced samples. The null saliva findings in the BPD cohort are best understood in the context of tissue specificity and cellular heterogeneity. Salivary DNAm is characterized by greater cellular heterogeneity than whole blood, as saliva captures variable proportions of epithelial cells and leucocytes whose DNAm profiles differ substantially [63,64]. This heterogeneity may obscure locus-specific associations detectable in blood, particularly in the absence of cell-type correction. These null results do not imply biological irrelevance of *SIAH3* DNAm in saliva; rather, they highlight the tissue- and cell-type specific nature of epigenetic associations (e.g., [65]). Since saliva samples were unavailable for the MDD and SAD cohorts, cross-cohort cross-tissue comparison of DNAm was not possible. In the MDD cohort, a significant effect of smoking status was observed, which is consistent with abundant evidence that tobacco smoke induces widespread DNAm changes (e.g., [66]). Further, a trend-level diagnosis × ACE interaction was detected, which reached significance only in the unadjusted sensitivity analysis but was not followed up on, given its lack of robustness to covariate adjustment. The pattern is consistent with ACE modulating *SIAH3* DNAm differently in MDD patients and HCs, though this interpretation requires replication. Possible contributing factors include limited sample size, interindividual variability, and confounders not modeled in the present study, such as medication use, psychiatric comorbidity, and symptom severity. In the SAD cohort, a diagnosis × ACE interaction emerged, as well. High ACE HC showed significantly lower *SIAH3* DNAm, whereas SAD patients exhibited no such ACE-related differences. Both the significant interaction and the post hoc pattern in HC were reflected in the sensitivity analyses. This may reflect an adaptive epigenetic response to early adversity in HC that may be disrupted in the presence of SAD pathology, though alternative interpretations, including differential influences of unmodeled clinical factors such as medication use, comorbidity, or symptom severity in SAD patients relative to HC, cannot be excluded. The SAD model explained substantially more variance in *SIAH3* DNAm than the other cohorts, confirming that diagnosis × ACE effects were most pronounced in SAD. The stronger IVW pooled estimate relative to the random-intercept GLMM estimate is consistent with the SAD cohort’s larger effects elevating the fixed-effects IVW estimate, while the random-intercept model more conservatively absorbs between-cohort heterogeneity. Previous research parallels our findings. Walker et al. (2016) [42] reported *SIAH3* hypermethylation as a prospective predictor of future mood disorder onset, although this finding did not survive multiple testing correction. Consistent with [43], a significant hypomethylation in high ACE HC was replicated, while additionally, a significant negative main effect of diagnosis and a significant diagnosis × ACE interaction were observed. The discrepancy, albeit using a subset of their participants herein, may arise from methodological differences, as Wiegand et al. (2021) [43] used a genome-wide approach, whereas our analysis targeted a specific region. Collectively, our findings suggest that *SIAH3* DNAm does not act as a uniform biomarker of ACE across aMD. Instead, ACE appears to exert disorder-specific effects, and aMD pathology may override or modify potentially adaptive epigenetic responses to early life stress. Although diagnosis × ACE interaction effects could not be assessed for the BPD cohort, similar trends may emerge, with HC showing lower *SIAH3* DNAm and patients diverging in an as-yet undetermined manner. Larger, balanced studies are required to confirm and extend these hypotheses.

Exploratory density-based stratification of blood *SIAH3* DNAm identified multimodal distributions in the BPD and MDD cohorts and in the pooled aMD sample. In the MDD cohort, pre-analytical variables did not predict mode membership, with no specific cell types differing across the identified modes. While this was initially consistent with MDD multimodality not being driven by cellular confounding, a subsequent formal dip test restricted to the cell-corrected MDD subset yielded non-significant results. Critically, the dip test applied to unadjusted DNAm values within this same subset was also non-significant, indicating that the marginal full-cohort multimodality signal is not recoverable at this subsample size regardless of cell-type adjustment. The adjusted-versus-unadjusted comparison within the subset is therefore uninformative about whether cellular composition drives the MDD mode structure, and the disappearance of the signal is best attributed to reduced statistical power. Well-powered analyses with cell-type data for the full cohort are needed to address this question. In contrast, the BPD cohort exhibited a significant association between blood storage duration and mode membership, which precludes confident biological interpretation of the BPD-specific mode structure. In the pooled aMD sample, apparent technical associations did not replicate in per-cohort analyses, most plausibly reflecting between-cohort differences in pre-analytical protocols. Partial associations with ACE exposure and CTQ subscales were identified, but no single demographic, clinical, technical, or cellular variable fully accounted for the observed groupings. Ultimately, while the lack of dip test significance in the cell-corrected subset necessitates an exploratory interpretation, the robust multimodality in the full MDD cohort remains an empirically observed pattern warranting further investigation. These exploratory findings motivate future, well-powered validation studies to definitively disentangle potential genetic, developmental, or epigenetic regulatory mechanisms from subtle cellular composition effects.

Interpretation of peripheral DNAm findings is inherently limited by tissue specificity [67]. While brain tissue cannot be examined in vivo, *SIAH3* has been associated with axonal degeneration and neurodegeneration [38,40], suggesting neuronal relevance. No data on *SIAH3* DNAm in human brain tissue was available, precluding direct inference. Several functional studies provide relevant context for interpreting *SIAH3* DNAm findings. [68] showed that *SIAH3* promoter hypermethylation reduces gene expression, reversible through DNA methyltransferase inhibition. SIAH3 was identified as a tumor suppressor that limits cell proliferation and mitochondrial respiration, leading to impaired cellular energy metabolism [68]. Moreover, *SIAH3* upregulation in mouse brains following maternal immune activation implicates it in stress-responsive molecular pathways, suggesting a potential mechanistic link of ACE exposure to psychiatric risk [44].

Speculatively, altered *SIAH3* expression could impact mitochondrial energy metabolism, a process increasingly recognized as central to stress biology and aMD pathophysiology [69,70]. In keeping with a potential functional link, a trend-level negative association between *SIAH3* blood DNAm and cell-type-adjusted gene expression values was observed in the SAD cohort. Within-group sensitivity analyses showed a directionally consistent negative association in both HC and SAD patients, arguing against a group-driven artefact. As this correlation did not reach statistical significance, is based on a single cohort, and concerns a gene at the low-expression noise floor in blood, the finding must be treated as exploratory and requires independent replication. Elevated mitochondrial DNA copy numbers, reflecting mitochondrial dysfunction [71], have been associated with both ACE and psychiatric outcomes [31], consistent with high psychiatric comorbidity in mitochondrial disease [72]. Collectively, these observations are consistent with a speculative model in which ACE-related *SIAH3* DNAm alterations may influence gene expression and mitochondrial function, potentially contributing to stress-related biological changes and aMD risk. However, direct mechanistic evidence in humans is currently lacking.

While promoter and first exon DNAm are typically repressive, intragenic DNAm can still influence expression through effects on transcription elongation or chromatin conformation [73,74,75,76]. Hence, *SIAH3* DNAm differences observed here may affect transcription, though direction and magnitude remain uncertain. Further, potential interactions with histone modifications or non-coding RNAs should be considered. While gene expression was assessed only for the SAD cohort, and mitochondrial activity was not directly measured, mechanistic interpretations remain speculative. Future studies should replicate and extend the SAD expression findings across diagnostic groups and investigate whether *SIAH3* exon 2 methylation is associated with mitochondrial dysfunction. If confirmed, such mechanisms could link ACE exposure to altered stress reactivity and energy metabolism, processes central to the shared psychological and somatic consequences of early adversity [77].

This study includes several limitations. In the BPD cohort, the unequal distribution of high ACE individuals prevented assessment of diagnosis × ACE interactions. Methodological differences in DNAm measurement may affect the comparability of absolute values of the SAD cohort with the BPD and MDD cohorts, though within-cohort analyses remain valid. Cell-type correction data were only available for the SAD cohort and a subset of the MDD cohort; no additional data were available to extend cell-type correction to the full BPD or MDD samples. This limits direct between-cohort DNAm comparisons. The Hartigan’s dip test applied to both the unadjusted and cell-type-adjusted DNAm values in the MDD subset yielded non-significant results, indicating that the full-cohort multimodality signal is not detectable at this subsample size regardless of adjustment. All multimodality findings are exploratory, and between-cohort DNAm comparisons should be interpreted with care. Clinical heterogeneity, including variation in medication use or symptom severity across participants, may have introduced variability not accounted for in the statistical models, and the potential influence of these factors on *SIAH3* DNAm cannot be excluded. Functional implications remain largely speculative. The cross-sectional design precludes causal inference, and KDE mode analyses should be considered exploratory, pending replication. Furthermore, pre-analytical variables significantly predicted mode membership in some cohorts, necessitating care in the biological interpretation of mode structure.

Future work should integrate DNAm, genotype, environmental, and expression data to clarify the sources and consequences of *SIAH3* DNAm variation across health and aMD states. Longitudinal and developmental studies are needed to test whether *SIAH3* DNAm mediates the lasting effects of ACE. Functional assays in neuronal models linking *SIAH3* to mitochondrial processes will be critical for establishing mechanistic relevance. Future studies should also harmonize cell-type correction across all cohorts and systematically account for clinical variation.

## 5. Conclusions

This study demonstrates that *SIAH3* DNAm is associated with ACE in a context-dependent, diagnosis-specific manner. While not a uniform biomarker of adversity, *SIAH3* appears sensitive to both trauma and aMD, possibly through mitochondrial regulation. Exploratory DNAm profiling revealed subgroups that were only trend-wise associated with trauma exposure and aMD, indicating that despite occasional pairwise differences, no variable assessed in this study consistently distinguished all modes. Notably, these marginal effects were observed not only for variables of clinical interest, but also for technical factors. These findings highlight both the promise of epigenetic stratification and the complexity of epigenetic regulation. Overall, the findings underscore the need for context-specific approaches to epigenetic biomarkers and support further mechanistic and translational exploration of *SIAH3* as a link between ACE, stress biology, and aMD. Future research integrating DNAm, gene expression, and mitochondrial functional assays will be essential to evaluate whether *SIAH3* represents a mechanistic link between early adversity and aMD risk.

## Figures and Tables

**Table 1:** Demographic and clinical data of the pooled aMD sample and HC, as well as respective clinical subgroups and their HC. Column *p* (W) shows *p*-values and W statistics of the Wilcoxon rank-sum test. * *p* = Fisher’s exact *p*-value. All data are shown as median (interquartile range (IQR)). Missing values or unassessed comparisons are indicated as NA.

	Pooled aMD Sample			BPD Cohort			MDD Cohort			SAD Cohort		
Variable	HC	aMD	*p* (W)	HC	BPD	*p* (W)	HC	MDD	*p* (W)	HC	SAD	*p* (W)
Sample size (*n*)	187	167		53	40		62	62		72	65	
Age [years]	25.00 (8.00)	28.00 (17.00)	0.0026 (12,565)	26.00 (9.00)	28.00 (10.25)	0.1266 (863)	26.00 (12.75)	39.00 (25.25)	<0.0001 (1098)	24.00 (6.00)	24.00 (9.00)	0.4836 (2502.5)
Sex female [%]	118 [63.10]	89 [53.94], NA = 2	0.0840 *	46 [86.79]	33 [82.50]	0.5740 *	44 [70.97]	36 [60.00], NA = 2	0.2536 *	44 [61.11]	45 [69.23]	0.3717 *
Smoker yes [%]	26 [15.48], NA = 19	50 [33.11], NA = 16	0.0003 *	4 [7.69], NA = 1	23 [57.50]	<0.0001 *	11 [17.74]	14 [23.33], NA = 2	0.5051 *	11 [20.37], NA = 18	13 [25.49], NA = 14	0.6433 *
CTQ score	32.00 (14.50)	43.00 (24.00)	<0.0001 (9387.5)	32.00 (9.00)	59.00 (25.25)	<0.0001 (123.5)	37.00 (21.75)	41.50 (17.00)	0.2187 (1619.5)	31.00 (15.00)	38.00 (22.00)	0.0053 (1693)
Emotional abuse score	7.00 (6.00)	11.00 (10.009)	<0.0001 (10,229)	7.00 (3.00)	17.00 (7.25)	<0.0001 (222)	8.00 (8.00)	9.00 (8.00)	0.4537 (1714)	7.00 (5.00)	9.00 (6.00)	0.0048 (1691)
Emotional neglect score	5.00 (2.00)	5.00 (5.00)	0.0027 (12,829.5)	8.00 (4.00)	17.00 (8.25)	<0.0001 (290)	5.00 (1.00)	5.00 (2.00)	0.1911 (1643.5)	5.00 (0.00)	5.00 (1.00)	0.0250 (1963.5)
Physical abuse score	5.00 (0.00)	5.00 (2.00)	<0.0001 (12,517.5)	5.00 (1.00)	9.50 (8.50)	<0.0001 (406.5)	5.00 (0.00)	5.00 (0.00)	0.8816 (1808.5)	5.00 (0.00)	5.00 (0.00)	0.0284 (2041)
Physical neglect score	7.00 (7.00)	11.00 (9.00)	<0.0001 (9716.5)	5.00 (2.00)	9.50 (6.25)	<0.0001 (402.5)	9.00 (10.00)	13.00 (9.00)	0.0218 (1387.5)	7.50 (8.00)	11.00 (9.00)	0.0030 (1654)
Sexual abuse score	5.00 (3.00)	7.00 (5.00)	<0.0001 (10,846.5)	5.00 (0.00)	10.00 (8.50)	<0.0001 (461.5)	6.50 (4.00)	7.50 (3.00)	0.2206 (1625.5)	6.00 (3.25)	6.00 (4.00)	0.2600 (2086.5)
ACE high [%]	58 [31.02]	91 [55.15], NA = 2	<0.0001 *	10 [18.87]	38 [95.00]	<0.0001 *	26 [41.94]	28 [46.67], NA = 2	0.7156 *	22 [30.56]	25 [38.46]	0.3704 *
BSL-23 score	NA	NA	NA	0.09 (0.15)	2.52 (0.96)	<0.0001 (1)	NA	NA	NA	NA	NA	NA
BDI score	NA	NA	NA	NA	NA	NA	2.00 (4.00)	21.00 (25.75)	<0.0001 (579.5)	NA	NA	NA
LSAS total score	NA	NA	NA	NA	NA	NA	NA	NA	NA	10.00 (14.50)	71.00 (45.00)	<0.0001 (106.5)
GSI score	NA	NA	NA	0.20 (0.28)	2.04 (0.68)	<0.0001 (0)	0.19 (0.39)	0.91 (1.04)	<0.0001 (857)	NA	NA	NA
PSDI score	NA	NA	NA	1.19 (0.34)	2.73 (0.63)	<0.0001 (6)	1.12 (0.43)	1.68 (0.80)	<0.0001 (940.5)	NA	NA	NA
PST score	NA	NA	NA	15.50 (16.00)	67.00 (15.50)	<0.0001 (0)	14.00 (20.75)	44.50 (39.75)	<0.0001 (873.5)	NA	NA	NA

**Table 2:** Epigenetic data of the pooled aMD sample, BPD, MDD, and SAD cohorts. All data is shown as median (IQR). Unassessed comparisons are indicated as NA.

Variable	Pooled aMD Sample		BPD Cohort		MDD Cohort		SAD Cohort	
	HC	aMD	HC	BPD	HC	MDD	HC	SAD
Blood DNAm CpG1 [%]	33.22 (42.44)	34.27 (34.34)	14.97 (28.40)	32.64 (26.35)	18.38 (24.10)	16.95 (26.89)	48.82 (37.08)	42.17 (35.67)
Blood DNAm CpG2 [%]	NA	NA	14.56 (30.38)	33.95 (29.43)	18.69 (27.51)	15.99 (28.03)	NA	NA
Blood DNAm CpG3 [%]	32.17 (44.80)	32.71 (38.38)	13.07 (27.20)	30.83 (28.06)	16.59 (25.56)	13.96 (27.46)	53.81 (34.92)	47.65 (30.50)
Blood DNAm CpG total [%]	33.75 (42.54)	35.16 (34.95)	14.11 (28.84)	32.48 (27.30)	17.80 (25.68)	15.67 (27.78)	51.11 (35.43)	44.35 (32.16)
Saliva DNAm CpG1 [%]	NA	NA	35.31(30.31)	38.70 (15.50)	NA	NA	NA	NA
Saliva DNAm CpG2 [%]	NA	NA	30.60 (28.06)	36.66 (16.06)	NA	NA	NA	NA
Saliva DNAm CpG3 [%]	NA	NA	30.17 (28.71)	35.62 (13.96)	NA	NA	NA	NA
Saliva DNAm total [%]	NA	NA	32.03 (29.14)	36.99 (15.12)	NA	NA	NA	NA

## Data Availability

The raw data are available in Table S1 of the Supplementary Materials.

## Supplementary Materials

The following supporting information can be downloaded at https://www.mdpi.com/article/10.3390/biom16070934/s1. Figure S1: *SIAH3* saliva DNAm in the BPD cohort is displayed with respect to diagnosis and ACE status. Figure S2: Cohort-stratified sensitivity analysis of the main effect of diagnosis on blood DNAm. Forest plot displaying effect ratios and corresponding 95% CIs for the primary diagnostic contrast (patients vs. HC) across independent cohorts (blue). Red diamonds indicate the fixed-effects meta-analytic summary derived via inverse-variance weighting (IVW) across all three cohorts, and the primary transdiagnostic GLMM estimate utilizing a (1|Cohort) random intercept framework. An effect ratio < 1.0 indicates lower mean DNAm levels in patients relative to HC; the dashed vertical line represents the null hypothesis (effect ratio = 1.0). All underlying models utilize a Gamma distribution with a log link function and the formula SIAH3 DNAm level ~ diagnosis × ACE + age + sex + smoking status (for BPD, the full interaction formula was used in the sensitivity analysis only; separate main-effect models were fitted in the primary analysis due to group imbalance). Figure S3: Spearman rank correlation between SIAH3 blood DNAm levels and cell-type-adjusted, DESeq2-normalized gene expression values. (A) shows the full cohort correlation, while (B) separates results for each diagnosis * ACE subgroup. N = sample size. ρ = Spearman’s rho. Table S1: Raw data. Table S2: Correlation analysis of *SIAH3* CpG sites for each clinical subgroup (BPD, MDD, and SAD cohorts). Table S3: Correlation analysis of *SIAH3* CpG sites for the pooled aMD sample. Table S4: Results of the GLMM with covariate adjustment for the pooled aMD sample (*SIAH3* DNAm level ~ diagnosis × ACE + age + sex + smoking status + (1|Cohort), AIC = 2736.5, dispersion estimate = 0.423; R^2^ = 0.0307). Table S5: Results of the post hoc tests of the GLMM with covariate adjustment for the pooled aMD sample. Table S6: Results of the GLMM without covariate adjustment for the pooled aMD sample (*SIAH3* DNAm level ~ diagnosis × ACE + (1|Cohort), AIC = 3033.5, dispersion estimate = 0.419, R^2^ = 0.0208). Table S7: Results of the post hoc tests of the GLMM without covariate adjustment for the pooled aMD sample. Table S8: Results of the GLM for diagnosis with covariate adjustment for the BPD (blood) cohort (*SIAH3* DNAm level ~ diagnosis + age + sex + smoking status, AIC = 742.7, dispersion estimate = 0.478, R^2^ = 0.0443). Table S9: Results of the GLM for diagnosis without covariate adjustment for the BPD (blood) cohort (*SIAH3* DNAm level ~ diagnosis, AIC = 746.1, dispersion estimate = 0.489, R^2^ = 0.0124). Table S10: Results of the GLM for ACE with covariate adjustment for the BPD (blood) cohort (*SIAH3* DNAm level ~ ACE + age + sex + smoking status, AIC = 741.6, dispersion estimate = 0.472, R^2^ = 0.0585). Table S11: Results of the GLM for ACE without covariate adjustment for the BPD (blood) cohort (*SIAH3* DNAm level ~ ACE, AIC = 743.5, dispersion estimate = 0.476, R^2^ = 0.0480). Table S12: Results of the GLM for diagnosis with covariate adjustment for the BPD (saliva) cohort (*SIAH3* DNAm level ~ diagnosis + age + sex + smoking status, AIC = 769.0, dispersion estimate = 0.226, R^2^ = 0.0167). Table S13: Results of the GLM for diagnosis without covariate adjustment for the BPD (saliva) cohort (*SIAH3* DNAm level ~ diagnosis, AIC = 771.7, dispersion estimate = 0.228, R^2^ = 0.0003). Table S14: Results of the GLM for ACE with covariate adjustment for the BPD (saliva) cohort (*SIAH3* DNAm level ~ ACE + age + sex + smoking status, AIC = 768.8, dispersion estimate = 0.226, R^2^ = 0.0190). Table S15: Results of the GLM for ACE without covariate adjustment for the BPD (saliva) cohort (*SIAH3* DNAm level ~ ACE, AIC = 771.0, dispersion estimate = 0.226, R^2^ = 0.0096). Table S16: Results of the GLM with covariate adjustment for the MDD cohort (*SIAH3* DNAm level ~ diagnosis × ACE + age + sex + smoking status, AIC = 1023.4, dispersion estimate = 0.412, R^2^ = 0.0849). Table S17: Results of the GLM without covariate adjustment for the MDD cohort (*SIAH3* DNAm level ~ diagnosis × ACE, AIC = 1022.3, dispersion estimate = 0.426, R^2^ = 0.0418). Table S18: Results of the GLM with covariate adjustment for the SAD cohort (*SIAH3* DNAm level ~ diagnosis × ACE + age + sex + smoking status, AIC = 962.6, dispersion estimate = 0.317, R^2^ = 0.1893). Table S19: Results of the post hoc tests of the GLM with covariate adjustment for the SAD cohort. Table S20: Results of the GLM without covariate adjustment for the SAD cohort (*SIAH3* DNAm level ~ diagnosis × ACE, AIC = 1251.2, dispersion estimate = 0.328, R^2^ = 0.1267). Table S21: Results of the post hoc tests of the GLM without covariate adjustment for the SAD cohort. Table S22: Characterization of the emerging DNAm modes in the pooled aMD sample. Numeric variables are shown as mean ± SD. Post hoc Dunn tests with Bonferroni correction were applied in case of significant Kruskal–Wallis comparisons. Non-significant Kruskal–Wallis comparisons are marked with ns. Categorical variables were characterized using Fisher’s exact test. * indicates Chi-square test instead of Fisher’s exact test for categorical variables. Table S23: Characterization of the emerging DNAm modes in the BPD (blood) cohort. Numeric variables are shown as mean ± SD. Post hoc Dunn tests with Bonferroni correction were applied in case of significant Kruskal–Wallis comparisons. Non-significant Kruskal–Wallis comparisons are marked with ns. Categorical variables were characterized using Fisher’s exact test. Table S24: Characterization of the emerging DNAm modes in the MDD cohort. Numeric variables are shown as mean ± SD. Post hoc Dunn tests with Bonferroni correction were applied in case of significant Kruskal–Wallis comparisons. Non-significant Kruskal–Wallis comparisons are marked with ns. Categorical variables were characterized using Fisher’s exact test. Table S25: List of supplementary figures and tables.

## Abbreviations

The following abbreviations are used in this manuscript:

ACE	Adverse Childhood Experiences
aMD	Adult Mental Disorders
BDI	Beck Depression Inventory
BPD	Borderline Personality Disorder
BSL-23	Borderline Symptom List
BDNF	Brain Derived Neurotrophic Factor
CI	95% Confidence interval
COMT	Catechol-O-Methyltransferase
CTQ	Childhood Trauma Questionnaire
DNAm	DNA methylation
EWAS	Epigenome-wide association study
EDTA	Ethylenediaminetetraacetic acid
EMMs	Estimated Marginal Means
GLMM	Generalized Linear Mixed-effects Model
GLM	Generalized Linear Model
GSI	Global Severity Index
HC	Healthy Control participants
IPDE	International Personality Disorder Examination
IQR	Interquartile range
IVW	Inverse-variance weighting
KDE	Kernel density estimation
LSAS	Liebowitz Social Anxiety Scale
MDD	Major Depressive Disorder
*MAOA*	Monoamine Oxidase A
PRKN	Parkin RBR E3 ubiquitin protein ligase
PSDI	Positive Symptom Distress
PST	Positive Symptom Total
PINK1	PTEN induced kinase 1
*SIAH3*	Seven in Absentia Homolog 3
SAD	Social Anxiety Disorder
SD	Standard deviation
SCID	Structured Clinical Interview for DSM-IV
SCL-90-R	Symptom Checklist-90-R
