# Supplementary material for: Investigating Peripheral SIAH3 DNA Methylation in Adult Mental Disorders in Relation to Adverse Childhood Experiences
Source: Biomolecules. 2026 Jun 23;16(7):934. doi: 10.3390/biom16070934 (PMC13407081; doi:10.3390/biom16070934)
Supplement: Supplementary file 1 [file biomolecules-16-00934-s001.zip › biomolecules-4344100_Supplement_Text_and_Figure.pdf]

Supplement: Investigating Peripheral *SIAH3* DNA Methylation in Adult Mental Disorders in relation to Adverse Childhood Events

Calculation of SIAH3 DNAm levels

The two technical replications of assessed DNAm in the BPD and MDD cohorts were averaged per CpG site and participant, resulting in a DNAm mean of the respective CpG sites. In the BPD cohort, this was performed separately for blood and saliva. Correlations of the CpG site values were tested using the Spearman rank correlation with Bonferroni correction for multiple testing (Table S2). After stable and highly significant correlations were confirmed, the DNAm values were averaged per participant for further statistical analyses and are in the following referred to as *SIAH3* blood and saliva DNAm level. In the SAD cohort, where only CpG site 1 and CpG site 3 data were available, *SIAH3* DNAm levels were calculated from only these two CpG sites and analyzed in the same way as the data from the BPD and MDD cohorts for downstream analyses. For the pooled aMD analysis, *SIAH3* DNAm levels were calculated as described for the SAD cohort after significant correlations were confirmed using Spearman rank correlation with Bonferroni correction for multiple testing (Table S3).

SIAH3 expression data and DNAm-expression correlation analysis

To explore the functional relevance of *SIAH3* DNAm, peripheral blood transcriptome data from the SAD cohort, previously published by [62] was utilized. Briefly, total RNA was extracted from whole blood collected in PAXgene Blood RNA tubes (samples with an RNA integrity number ≥ 7 were retained) and subjected to 3’ RNA-sequencing using the QuantSeq 3’ mRNA-Seq Library Prep Kit (Lexogen), with sequencing performed at a depth of approximately 10 million 100-bp reads on an Illumina NovaSeq platform at the NGS Competence Center Tübingen (NCCT). Read preprocessing, quality trimming and alignment to the human reference genome (GRCh38.104) were performed using the Lexogen pipeline incorporating BBTools (bbduk) and the STAR aligner. Gene-level counts were normalized using DESeq2’s median-of-ratios method. To account for the influence of blood cell type composition on gene expression, cell type proportions were estimated from TPM-normalized counts, and all gene counts were subsequently adjusted to the estimated cell type ratios using a linear regression-based deconvolution approach, as described in detail in [62]. The expression data used in the present analysis therefore reflect cell-type-adjusted, DESeq2-normalized values. It should be noted that *SIAH3* is lowly expressed in peripheral blood: the underlying raw read counts fell below the ≥ 20-count-per-sample detection threshold applied in the original transcriptome-wide analyses by [62], which consequently excluded *SIAH3*. Cell-type adjusted, DESeq2-normalized expression values for *SIAH3* had a median of 2.38 (range: -1.65-39.91), with 13.1 % of samples yielding negative adjusted values, reflecting variability at the low-expression noise floor. The present analysis therefore uses these low-count values in an exploratory capacity only and results need to be treated with appropriate caution. The association of *SIAH3* blood DNAm and cell-type adjusted gene expression values was assessed using Spearman rank correlation, as both variables showed non-normal distributions (Shapiro-Wilk test).

GLMM and GLM analyses

For the pooled aMD sample, diagnosis, ACE and their interaction were modeled as fixed effects, with age, sex, and smoking status as covariates, while cohort was included as a random intercept to account for between-cohort variability (*SIAH3 DNAm level ~ diagnosis*ACE + age + sex + smoking status + (1|Cohort)*). While no random effects were included in the per cohort analyses, rendering these models technically generalized linear models (GLMs) rather than mixed-effects models, the same package was chosen for its flexibility in handling right-skewed distributions, and to ensure comparability across analyses. As in the GLMM, Gamma distribution and log link were applied. Due to group imbalance in ACE exposure within the BPD cohort (Table 1, Figure 1, Figure S1), interaction effects between diagnosis and ACE could not be modeled. Instead, separate models were fitted using the formulae *SIAH3 DNAm level ~ diagnosis + age + sex + smoking status* and *SIAH3 DNAm level ~ ACE + age + sex + smoking status*, for both blood and saliva DNAm. For the MDD cohort, the model included *SIAH3*

*DNAm level ~ diagnosis*ACE + age + sex + smoking status*, and for the SAD cohort, the formula *SIAH3 DNAm level ~ diagnosis*ACE + age + sex + smoking status* was applied. All group comparisons were repeated without covariate (age, sex, smoking status) inclusion as sensitivity analyses and reported in the supplement.

KDE analysis

Since preliminary data inspection revealed groupings of DNAm across cohorts and diagnoses, exploratory kernel density estimation (KDE) analysis was performed. This assumption-free, mathematically defined approach was selected given limited sample sizes, especially for the cohort-specific analyses, and low data dimensionality. For downstream characterization, all available variables were compared across DNAm modes to determine whether DNAm-defined states were associated with phenotypic, clinical, or technical factors. For a subset of 94 participants of the MDD cohort (57 HC and 37 MDD patients), cell counts of neutrophils, lymphocytes and monocytes were available. To test whether the inability to correct for cell counts (due to the lack of cell counts for a part of the MDD and the full BPD cohorts) might have driven the groupings, Spearman's correlation between adjusted and unadjusted DNAm values was assessed, and cell counts were included in the characterization approach (KDE based on unadjusted DNAm values of the full MDD cohort).

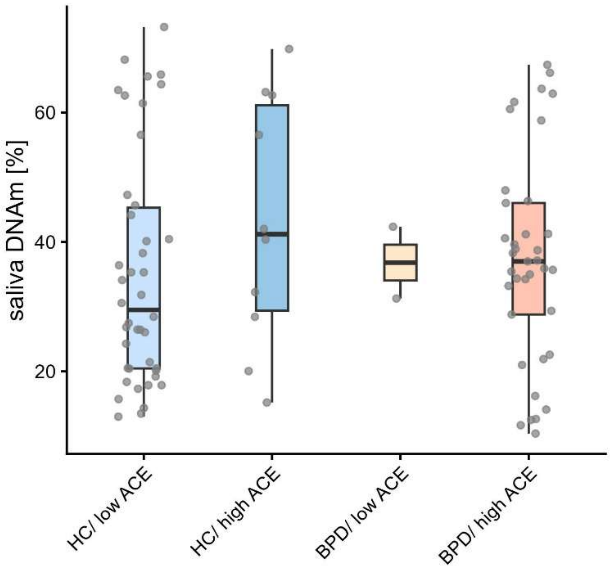

Figure S1: *SIAH3* saliva DNAm in the BPD cohort is displayed with respect to diagnosis and ACE status.

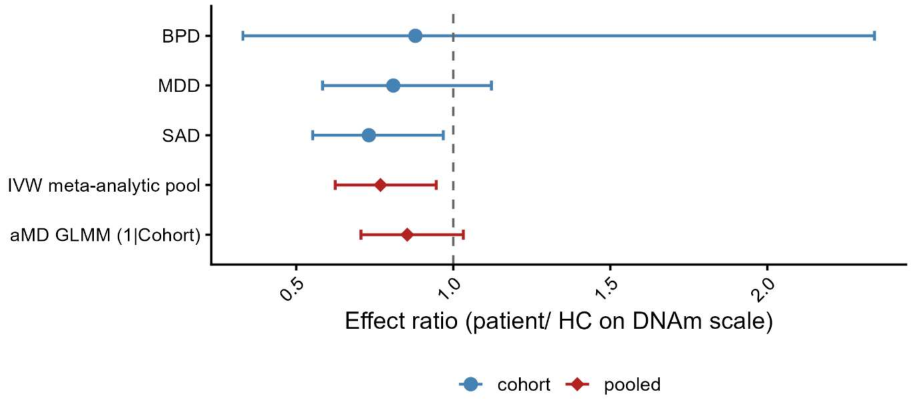

Figure S2: Cohort-stratified sensitivity analysis of the main effect of diagnosis on blood DNAm.. Forest plot displaying effect ratios and corresponding 95 % CIs for the primary diagnostic contrast (patients vs. HC) across independent cohorts (blue). Red diamonds indicate the fixed-effects meta-analytic summary derived via inverse-variance weighting (IVW) across all three cohorts, and the primary transdiagnostic GLMM estimate utilizing a *(1|Cohort)* random intercept framework. An effect ratio < 1.0 indicates lower mean DNAm levels in patients relative to HC; the dashed vertical line represents the null hypothesis (effect ratio = 1.0). All underlying models utilize a Gamma distribution with a log link function and the formula *SIAH3 DNAm level ~ diagnosis × ACE + age + sex + smoking status* (for BPD, the full interaction formula was used in the sensitivity analysis only; separate main-effect models were fitted in the primary analysis due to group imbalance).

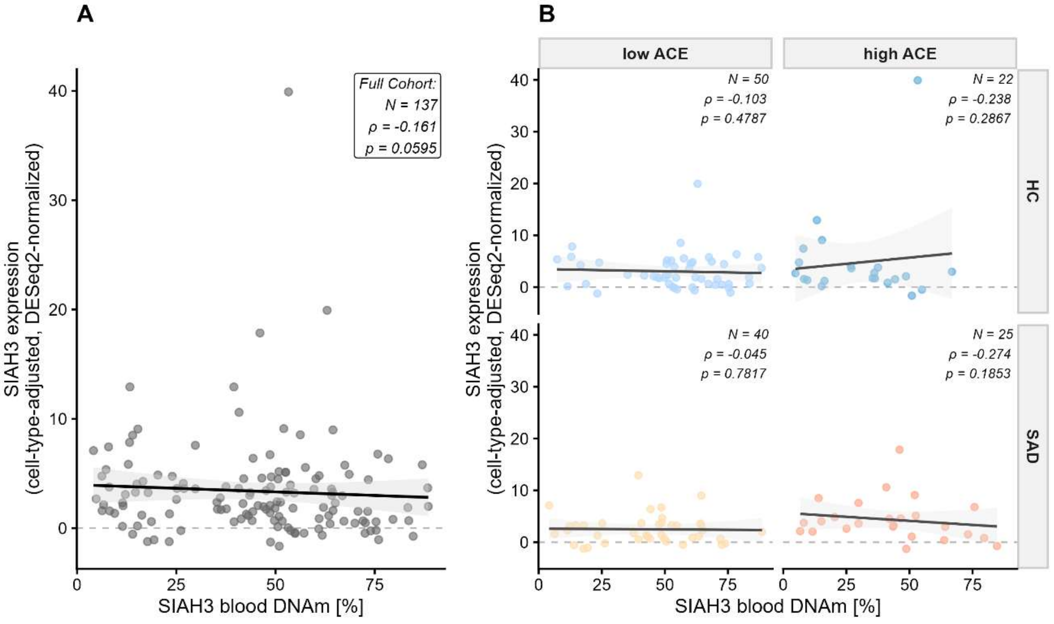

Figure S3: Spearman rank correlation between *SIAH3* blood DNAm levels and cell-type-adjusted, DESeq2-normalized gene expression values. A) shows the full cohort correlation, while B) separates results for each diagnosis*ACE subgroup. N = sample size. ρ = Spearman's rho.
